# From metabolic reprogramming to immune homeostasis: the bidirectional regulatory network of HIF-1*α*/HK2 and Th17/Treg in gout

**DOI:** 10.3389/fmed.2026.1967105

**Published:** 2026-10-30

**Authors:** Lianggang Wei, Jianping Gan, Chunhui Cao, Jing Wang, Yi Ren, Shasha Wang, Min Zhou

**Affiliations:** Department of Rheumatology and Immunology, Chongqing Traditional Chinese Medicine Hospital, Chongqing, China

**Keywords:** glycolysis, gout, HIF-1*α*, HK2, metabolic reprogramming, NLRP3 inflammasome, Th17/Treg balance

## Abstract

Gout is a metabolic inflammatory disease whose pathogenesis involves the complex interaction between metabolic reprogramming and immune homeostasis imbalance. This review focuses on the bidirectional network through which the HIF-1*α*/HK2 axis regulates the Th17/Treg balance via glycolytic reprogramming. Monosodium urate (MSU) crystals drive enhanced aerobic glycolysis in macrophages through the GLUT1-JNK-HIF-1*α* signaling axis, where hexokinase 2 (HK2), as a metabolic checkpoint, connects glycolysis with NLRP3 inflammasome activation and neutrophil extracellular trap (NETosis)-induced M1 polarization. The glycolytic metabolic bias promotes the differentiation of T helper 17 (Th17) cells while inhibiting the generation of regulatory T (Treg) cells. In turn, cytokines derived from Th17/Treg cells reversely regulate the metabolic status of antigen-presenting cells, forming a feedback loop for inflammation amplification or resolution. Based on this network model, intervention strategies targeting the HIF-1*α*/HK2/mTOR glycolytic axis and metabolic checkpoints (such as HK2, PKM2), including chaetocin, rapamycin, and various natural products, have shown therapeutic potential for restoring immune homeostasis. Current evidence mainly comes from animal models and *in vitro* experiments. In the future, it is necessary to use single-cell multi-omics technology to resolve the heterogeneity of the immunometabolic landscape, and to verify the dynamic regulatory mechanism of this network in the self-limiting resolution of gout inflammation.

## 1 Introduction

Gout is a metabolic inflammatory disease caused by monosodium urate (MSU) crystal deposition, with pathogenesis driven by the interaction of genetic susceptibility, metabolic disturbance and immune microenvironment disorder (1). Standard urate-lowering therapies can stabilize serum uric acid levels but fail to prevent recurrent flares in some patients, indicating that simple urate reduction cannot correct the underlying immunometabolic dysfunction of gout (2).

In the gout inflammatory microenvironment, MSU crystals induce aerobic glycolysis enhancement (the Warburg effect) in macrophages, which depends on the stable expression of hypoxia-inducible factor-1*α* (HIF-1*α*) (1, 2). HIF-1*α* upregulates glycolytic genes to meet inflammatory cell energy demands and amplifies inflammation by promoting NLRP3 inflammasome activation and IL-1β secretion. T cell differentiation is strictly regulated by metabolic signals: glycolysis promotes pro-inflammatory Th17 cell differentiation, while fatty acid oxidation (FAO) supports regulatory T (Treg) cell generation. The Th17/Treg imbalance and elevated pro-inflammatory factors such as IL-17 in gout patients confirm the pivotal role of the metabolism-immune axis in disease pathogenesis (3, 4).

This review focuses on the regulatory mechanism of the HIF-1*α*/HK2 axis in gout inflammation. We systematically elaborate how this axis modulates Th17/Treg balance via metabolic reprogramming to govern the initiation, persistence and resolution of gouty inflammation, aiming to provide a novel theoretical basis for gout pathogenesis and precision metabolic targeted therapy.

## 2 HIF-1*α*/HK2 axis dominates metabolic reprogramming in gout

### 2.1 MSU crystals induce HIF-1*α*-dependent glycolytic reprogramming

MSU crystals, the core inflammatory trigger of gout, drive macrophage metabolic reprogramming by upregulating glucose transporter 1 (GLUT1) expression to enhance glucose uptake and aerobic glycolysis (5). This metabolic switch is an essential upstream event for NLRP3 inflammasome activation and IL-1β release; glucose deprivation or GLUT1 inhibition significantly blocks MSU-induced inflammation (5). Mechanistically, MSU crystals activate the JNK signaling pathway (rather than p38) to sustain JUN transcription factor activity, which initiates the transcription of glycolytic enzymes and metabolic transporters independent of inflammasome activation, enabling direct metabolic reprogramming of macrophages (6).

HIF-1*α* acts as a core hub linking metabolism and inflammation. Single-cell transcriptomics has verified enriched hypoxia pathways and activated HIF1A transcription in peripheral monocytes of gout patients, which controls IL-1β production (7). Stabilized HIF-1*α* upregulates key glycolytic enzymes (HK2, PKM2) and GLUT1, forming a positive feedback loop of glycolysis amplification and inflammatory activation. Additionally, altered hepatic fatty acid metabolism activates HIF-1*α*, upregulating uric acid synthases to link systemic metabolic disorder and local joint inflammation. The GLUT1-JNK-HIF-1*α* signaling axis thus constitutes the core molecular basis of acute gout flares.

### 2.2 HK2 bridges glycolysis and inflammatory activation

HK2 catalyzes the first irreversible step of glycolysis, phosphorylating glucose to glucose-6-phosphate, and serves as a key regulator of glycolytic flux in inflammatory cells. MSU-induced neutrophil extracellular traps (NETs) promote macrophage M1 polarization and NLRP3 activation via targeting HK2, while HK2 silencing completely abolishes NETs-mediated metabolic and inflammatory abnormalities, confirming that HK2 is the key effector of NETs-induced pro-inflammatory responses (8).

HIF-1*α* directly transcriptionally regulates HK2 expression. The fungal metabolite chaetocin alleviates MSU-induced joint inflammation by inhibiting HIF-1*α*/HK2 expression and reducing NLRP3-dependent IL-1β secretion (9). Lcn2 deficiency suppresses glycolysis via HK2 downregulation and reduces mitochondrial ROS, thereby inhibiting NLRP3 activation (10). Notably, HK2 exerts tissue-specific metabolic regulation: renal cathepsin B upregulates HK2/PKM2 to enhance glycolysis, further inducing urate reabsorption transporters and aggravating hyperuricemia (11). Anchored on the outer mitochondrial membrane, HK2 directly senses metabolic stress, acting as a key metabolic checkpoint linking NETosis and macrophage functional polarization.

### 2.3 Metabolic reprogramming couples with NLRP3 inflammasome activation

Enhanced glycolysis triggers NLRP3 inflammasome activation through multiple pathways. Excessive glycolysis causes mitochondrial stress, increases ROS production, induces ATP depletion and K + efflux, and disrupts mitochondrial membrane potential to release mitochondrial DNA, all of which are classic upstream signals for NLRP3 inflammasome assembly and activation (2).

Mitochondrial pyruvate carrier (MPC) inactivation is another key coupling link. MPC inhibition shifts pyruvate metabolism from oxidative phosphorylation to lactic acid fermentation, enhances glycolysis, and potentiates macrophage inflammatory responses to MSU crystals (12). Clinically, the MPC inhibitor pioglitazone increases gout risk in diabetic patients, these findings collectively provide convergent support for the metabolism-inflammation link in gout. It should be noted that the clinical association is observational in nature, and further causal studies are needed to rule out potential confounding factors. Natural products including cucurbitacin B and kaempferol exert anti-gout effects by inhibiting glycolytic enzyme expression, blocking NLRP3 assembly and restoring immune balance (4, 13), highlighting the therapeutic potential of targeting this coupling node.

## 3 Th17/Treg imbalance mediates gout immune homeostasis collapse

### 3.1 Clinical characteristics of Th17/Treg imbalance in gout

Clinical studies have confirmed consistent Th17/Treg imbalance in gout patients: peripheral Th17 proportion increases, Treg proportion decreases, and the elevated Th17/Treg ratio is positively correlated with disease activity. A 126-case cohort study showed early-onset gout is dominated by Th17 elevation, while late-onset gout is characterized by Treg reduction, with disordered cytokine networks involving IL-2, IL-6, IL-17 and TNF-α participating in immune imbalance regulation (14).

This immune disorder presents dynamic phase-specific changes. In acute gout attacks, serum IL-17 rises sharply with disease aggravation and decreases during remission, mainly derived from *γδ*T cells in the early stage (15). Single-cell sequencing revealed enriched pro-inflammatory Th1/Th17 cells and activated effector T cells in synovial fluid of acute gout patients, with the highest inflammatory activity, confirming that Th17/Treg imbalance is a core pathological feature of gout with stage-specific differentiation characteristics (16).

### 3.2 Molecular mechanisms underlying Th17/Treg dysregulation

Multiple molecules regulate Th17/Treg balance in gout. Galectin-9 alleviates MSU-induced joint inflammation by reducing infiltrated inflammatory monocytes and correcting Th17/Treg ratio disorder (17). Uric acid directly promotes Th17 differentiation and renal inflammatory infiltration via activating the NF-*κ*B pathway (18). Mechanistically, inflammasome-activated IL-1β cooperates with IL-6/IL-23 to activate IL-1R signaling and drive Th17 cell expansion, forming a self-amplifying inflammatory loop (19). In contrast, TGF-*β*1 maintains Treg stability and Foxp3 expression by inhibiting mTORC1 glycolytic signaling and enhancing PP2A/STAT5 activity, which is essential for immune homeostasis maintenance (20).

### 3.3 Targeted intervention strategies for Th17/Treg imbalance

Current interventions targeting Th17/Treg imbalance include biological agents, immunosuppressants and natural products. IL-17A neutralizing antibodies alleviate gout arthritis by selectively inhibiting Th17 cells without affecting Treg function (21). Rapamycin remodels T cell metabolism by blocking Th17 glycolysis and promoting Treg fatty acid oxidation to restore immune balance (22).

Multiple traditional Chinese medicine active ingredients and compounds exert immunometabolic regulatory effects. Celastrol, kaempferol and quercetin correct Th17/Treg imbalance by regulating STAT3/STAT5 phosphorylation and glycolytic flux, reducing inflammatory factor release (4, 23, 24). Classic compounds including Sanmiao Pill and Simiao Pill inhibit the STAT3/ROR*γ*t and P2X7/IL-17 pathways to lower uric acid and relieve inflammation (25). However, most current evidence is from *in vitro* and animal experiments, and clinical translation and precise target verification remain insufficient.

## 4 Bidirectional regulatory network of HIF-1*α*/HK2 and Th17/Treg

### 4.1 Glycolysis determines T cell differentiation fate

HIF-1*α*-mediated glycolytic reprogramming has been identified as a key regulator of CD4+ T cell differentiation, with emerging evidence suggesting that HK2 may contribute to this process by supporting glycolytic flux. Stabilized HIF-1*α* upregulates GLUT1/HK2 to enhance glycolysis, activates mTOR/STAT3 signaling and upregulates ROR*γ*t, thereby promoting Th17 differentiation. Meanwhile, glycolytic activation inhibits Foxp3 stability and Treg generation, while Treg maintenance relies on TGF-*β*1-mediated glycolysis suppression and fatty acid oxidation enhancement (20). The transcription factor Bcl-6 further antagonizes HIF-1*α*-driven glycolysis to maintain T cell resting state (26).

Targeting glycolysis reverses immune imbalance: rapamycin and celastrol inhibit HIF-1*α* expression and Th17 glycolysis, promote Treg fatty acid oxidation, and restore Th17/Treg homeostasis (22, 24), verifying that the HIF-1*α*/HK2 glycolytic axis is a key metabolic checkpoint for gout immune regulation.

### 4.2 Immune feedback regulates metabolic reprogramming

Th17/Treg-derived cytokines form a feedback loop to control macrophage metabolism. Th17-secreted IL-17 activates macrophage NF-*κ*B/MAPK pathways, upregulates glycolytic enzymes, sustains pro-inflammatory metabolic phenotype, and continuously promotes NLRP3 activation and IL-1β secretion (2). Conversely, Treg-derived IL-10 and TGF-*β*1 inhibit mTOR/HIF-1*α* activity, suppress glycolysis, shift metabolism to oxidative phosphorylation, and induce tolerogenic immune phenotypes (27).

In acute gout, IL-17-mediated glycolytic positive feedback dominates to amplify inflammation; during disease remission, increased Treg-derived anti-inflammatory cytokines enhance metabolic negative feedback, driving inflammation self-resolution. This dynamic immune-metabolic crosstalk may provide a potential mechanistic framework that could account for the self-limiting nature of acute gout flares, which warrants further validation (2).

### 4.3 Integrated model of bidirectional regulatory network and the hypothesis of self-limiting gout inflammation

Based on the aforementioned interaction mechanism, we propose the HIF-1*α*/HK2-Th17/Treg bidirectional regulatory network model to explain gout inflammation's initiation, amplification, and self-limiting resolution (Figure 1). MSU crystals activate HIF-1*α* and upregulate glycolytic enzymes such as HK2, driving macrophage polarization to pro-inflammatory phenotype, and providing a metabolic microenvironment for Th17 cell differentiation (1, 2). Enhanced glycolysis directly supports Th17 cell function, and weakens Treg cell generation by inhibiting fatty acid oxidation, forming a pro-inflammatory positive feedback (22). However, with inflammation progression, metabolic substrate depletion, hypoxia signal changes, and anti-inflammatory cytokines derived from Treg can reversely inhibit glycolysis and promote oxidative phosphorylation, driving inflammation resolution, which may be one of the mechanisms for acute attacks’ self-limiting nature (2). Emerging evidence suggests that HK2 and PKM2 may act as key regulatory nodes in the immunometabolic network of gout, with their dysregulation linked to abnormal macrophage metabolism and renal uric acid handling (11, 28).

**Figure 1:**
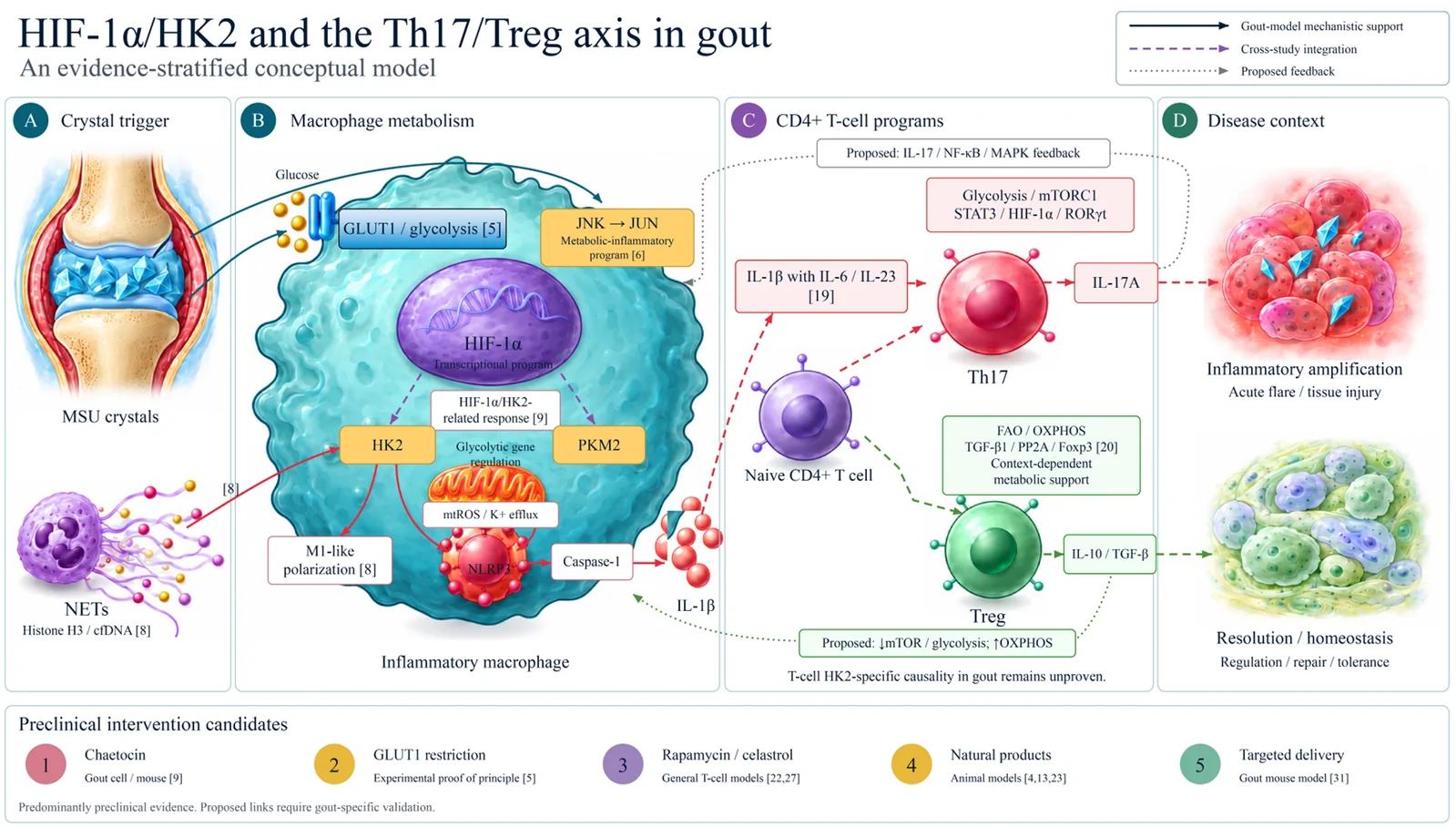
HIF-1α/HK2 and Th17/Treg axis in gout.

This model still has evident research gaps: the temporal dynamic correlation of HIF-1*α*/HK2 axis activity and Th17/Treg ratio remains unclear; clinical sample verification is insufficient; and non-specific systemic glycolysis inhibition may cause off-target effects (2, 29).

## 5 Targeted immunometabolic therapeutic strategies for gout

### 5.1 HIF-1*α*/HK2 axis-targeted drug intervention

Chaetocin exerts anti-gout activity by dual inhibition of HIF-1*α*/HK2 glycolytic pathway and NLRP3 inflammasome assembly, reducing joint inflammation in mouse models (9). GLUT1-targeted glycolysis blockade also effectively suppresses MSU-induced inflammation, providing a theoretical basis for metabolic checkpoint-targeted drug development (5). Emerging nanodelivery technology optimizes metabolic targeted therapy: macrophage membrane-coated melatonin liposomes target inflamed joints, inhibit glycolysis, and promote M2 macrophage polarization, achieving precise anti-gout effects with reduced systemic side effects (30).

### 5.2 Natural products and traditional Chinese medicine therapy

Flavonoids including kaempferol, quercetin and mangiferin improve gout via multi-target regulation of IL-17, NLRP3 and HIF-1 pathways to correct immune and metabolic disorders (4, 23, 31, 35). Cucurbitacin B and plant polysaccharides inhibit NLRP3 assembly to reduce inflammatory cytokine secretion (13, 32). Traditional Chinese medicine formulas such as Guizhi Shaoyao Zhimu Decoction and Quzhuo Tongbi Prescription regulate intestinal flora, inhibit glycolysis and restore immune homeostasis, showing unique multi-target therapeutic advantages (33).

### 5.3 Challenges and prospects of metabolic checkpoint therapy

Targeting immune metabolic checkpoints offers a novel strategy for gout treatment that enables simultaneous intervention in both inflammation and immune dysregulation. The key nodes of glycolysis serve as the shared metabolic basis for MSU-induced NLRP3 activation and Th17 pro-inflammatory differentiation (5). In preclinical models, glycolysis inhibition has been shown to promote Treg generation and restore Th17/Treg balance in T cells, while also reducing pro-inflammatory activation of macrophages by normalizing their metabolic state (20). However, the universal conservation of glycolytic pathways leads to potential off-target risks of systemic intervention. Glycolysis is a fundamental metabolic pathway for normal immune cell function. Broad systemic inhibition of glycolysis may lead to unintended immunosuppression or other adverse effects, which highlights the need for cell-type specific targeting strategies. Cell-specific targeted delivery systems and selective checkpoint inhibitors are the key to solving this problem (28). Metabolomics studies have confirmed distinct serum metabolic signatures in gout patients at different stages, providing novel predictive biomarkers and intervention targets (34). It should be noted that most of the current findings are derived from *in vitro* cell experiments and animal models, while clinical evidence for these therapeutic strategies is still limited.

## 6 Discussion

Accumulating evidence suggests that HIF-1*α*/HK2 axis-mediated glycolytic reprogramming may be an important contributor to Th17/Treg imbalance in gout, representing a key part of our proposed integrated regulatory model. Gout is no longer simply defined as a hyperuricemic metabolic disease, but a maladaptive immunometabolic disorder caused by crystal-induced metabolic reprogramming and immune homeostasis collapse (1, 2).

Current research is limited by insufficient clinical translational evidence and unclear metabolite-mediated metabolism-epigenetic crosstalk. The long-term safety of glycolysis-targeted drugs also needs further verification. Future research should apply single-cell multi-omics technology to resolve the dynamic immunometabolic landscape of gout, develop precise nanotherapy targeting macrophage metabolic checkpoints, and explore the metabolic regulatory mechanism of inflammation self-resolution. Elucidating the HIF-1*α*/HK2-Th17/Treg network will provide new insights for the precise clinical prevention and treatment of gout.

It is important to note that while the individual components of this network have been independently verified, direct causal evidence linking HIF-1*α*/HK2 activity to Th17/Treg imbalance specifically in human gout is still lacking. Future studies with temporal resolution and clinical validation are needed to confirm the causal relationship between these factors.
